# Immunohistochemical analysis of extracellular signal-regulated kinase expression in mature and immature bulls’ testes and epididymides

**DOI:** 10.17221/34/2023-VETMED

**Published:** 2023-06-28

**Authors:** Sungwoong Jang, Changjin Yun, Bohye Kim, Sohi Kang, Jeongmin Lee, Sohee Jeong, Yongho Cho, Sung-Ho Kim, Chang-Min Lee, Changjong Moon, Joong-Sun Kim

**Affiliations:** College of Veterinary Medicine and BK21 Plus Project Team, Chonnam National University, Buk-gu, Gwangju, Republic of Korea; Sungwoong Jang and Changjin Yun contributed equally to this work

**Keywords:** bovine, extracellular signal-regulated kinase, histology, testis

## Abstract

Extracellular signal-regulated kinase (ERK) has been implicated in mammalian testicular and epididymal development. This study aimed to investigate ERK expression in the immature and mature testes and epididymides of bulls. We evaluated ERK expression using immunoblot analysis and immunohistochemistry. Immunoblot analysis revealed that immature bull testes and epididymides had higher phosphorylated ERK (pERK) expression than mature bull testes and epididymides. pERK immunoreactivity was higher in immature epididymides than in immature testes. pERK was localised mostly in spermatogonia, undifferentiated sustentacular (Sertoli) cells, and interstitial (Leydig) cells in immature testes, as well as in some spermatocytes and spermatids in mature testes. In immature epididymides, the body and tail had higher pERK expression than the head, whereas pERK was broadly distributed throughout the stereocilia, basal cells, and connective tissues. pERK distribution in the head of mature epididymides was similar to that in immature epididymides, whereas few connective tissue cells were expressed in the body and tail of mature epididymides. Collectively, these results suggest that ERK is expressed in the testis and epididymis of immature and mature bulls with varying intensities, and the role of ERK in male reproductive organs may include the specific function of its development.

The development of organ systems requires complex cell signalling interactions to initiate the differentiation and development of various cell and tissue types essential for organ function (Windley and Wilhelm 2015). The mitogen-activated protein kinase (MAPK) signalling pathway is one of the crucial signal transduction systems and is involved in various cellular processes, including cell proliferation, differentiation, adhesion, migration, apoptosis, and response to environmental stimuli (Cargnello and Roux 2011; Busca et al. 2016). Mammalian cells comprise three MAPK units: c-Jun N-terminal kinase, p38, and the extracellular signal-regulated kinase (ERK) subfamily (Roux and Blenis 2004). Among these MAPK cascades, the role of ERK1/2 cascade in steroidogenesis has been recently documented both *in vivo* and *in vitro* (Crepieux et al. 2001). In mammalian cells, the ERK1/2 cascade constitutes a crucial signalling pathway that regulates cell proliferation, differentiation, and apoptosis and is regarded as a key factor influencing the development of the testis and epididymis as well as the maintenance of cell survival in the adult reproductive system (Xu et al. 2010; Xu et al. 2011; Windley and Wilhelm 2015). Although MAPK pathways, especially ERK, play an essential role in regulating mammalian reproductive functions by induction of the gonadotropins luteinizing hormone (LH) and follicle-stimulating hormone, studies of ERK expression during the development of the male reproductive system are limited (Shupe et al. 2011; Kanasaki et al. 2012).

In this study, we aimed to obtain fundamental data on the effect of the ERK1/2 cascade in the male reproductive organ maturation and function–acquisition processes by comparing ERK1/2 expression patterns between immature calves and adult bulls.

## MATERIAL AND METHODS

### Animals and tissue sampling

The testes and epididymides of three mature (24 months) and immature (5 months) bulls (Hanwoo, Korean native cattle) were obtained from a local farm and abattoir. The procedures and protocols followed in the present study were according to the Institutional Animal Care and Use Committee of the Chonnam National University (Approval No. CNU IACUC-YB-2022-16). The immature samples were collected during the planned castration process undertaken for improving meat quality. For histological analysis, the samples were fixed for one week in 10% buffered formalin. The contralateral testis and epididymis were snap-frozen and stored at −70 °C until western blotting.

### Histological analysis of bull testis and epididymis

After fixation, samples were trimmed and rinsed for 2 h in running tap water, then dehydrated in a series of ethanol concentrations ranging from 70% to 100%, cleared in xylene, embedded in paraffin, and sectioned into 4-μm-thick slices. After deparaffinization, haematoxylin and eosin staining were performed on the sections.

All samples used in this study were confirmed to have no histopathological changes, including inflammation, and the epididymis was divided into three regions (the head, body, and tail) based on histological features, such as differences in epithelial thickness.

### Immunohistochemistry

For antigen retrieval, the paraffin-embedded tissue sections were heated in citrate buffer (0.01 M, pH 6.0) for 1 h at 90 °C after deparaffinization. To suppress endogenous peroxidase activity after cooling, the sections were treated with 0.3% hydrogen peroxide in distilled water for 20 minutes. The sections were first incubated in normal goat serum for 1 h (Vectastain Elite ABC kit; Vector Laboratories, Burlingame, CA, USA) to avoid nonspecific binding.

Then, sections were exposed to rabbit polyclonal anti-pERK1/2 and anti-ERK1/2 antibodies (both 1 : 1 000; Cell Signaling Technology, Danvers, MA, USA) for an overnight reaction at 4 °C.

To obtain the negative control, we excluded the step on primary antibodies from the protocol. In all procedures, phosphate-buffered saline was used to wash the sections. The sections were exposed to biotinylated goat anti-rabbit immunoglobulin G (Vectastain Elite ABC kit) for 1 h, followed by incubation with the avidin–biotin peroxidase complex (Vectastain Elite ABC kit) to activate immunoreactivity.

A diaminobenzidine substrate kit (DAB Substrate Kit SK-4100; Vector Laboratories, Burlingame, CA, USA) was used to visualise the immunoreactivity, followed by haematoxylin counterstaining, dehydration, and mounting of the sections. The intensity of staining was graded from 0 to 3 as follows: negative (0), weakly positive (1), moderately positive (2), and strongly positive (3) (Ahmad et al. 2010).

### Western blotting

Tissue samples from the testis and three regions of the epididymis were homogenised using a lysis buffer, which contained 120 mM NaCl, 40 mM Tris, 2 mM Na_3_VO_4_, 0.1% Nonidet 40, 10 g/ml aprotinin, 1 mM phenylmethylsulfonyl fluoride, and 10 g/ml leupeptin. The supernatant was collected after 20 min of centrifugation at 30 000 *g*. The supernatant’s protein content was determined for the immunoblot analysis using the Bradford protein assay (Bio-Rad, Hercules, CA, USA). Equal samples of 20 g were loaded per lane, subjected to SDS-PAGE, and then blotted onto nitrocellulose membranes (Schleicher and Schuell, Keene, NH, USA) using standard methods. Blotting membranes were incubated with 5% non-fat milk in Tris-buffered saline (TBS; 10 mM Tris-HCl, pH 7.4, and 150 mM NaCl) for 1 h to block any remaining binding sites. The membranes were then incubated with rabbit polyclonal anti-pERK1/2 and anti-ERK1/2 antibodies for 2 hours.

TBS containing 0.1% Tween 20 was used to wash the blots three times before they were treated with horseradish peroxidase-conjugated anti-rabbit antibody (1 : 10 000 dilution; Thermo Fisher Scientific, Inc., Rockford, IL, USA) for 1 hour. The enhanced chemiluminescence kit (SuperSignal^®^ West Pico; Thermo Fisher Scientific, Inc., Rockford, IL, USA) was used to develop membranes for 1 min according to the producer’s guidelines, and then they were subjected to an X-ray as directed by the manufacturer (Agfa Gevaert N.V., Mortsel, Belgium). The membranes were stripped and re-probed using a monoclonal anti-actin antibody (1 : 10 000; Sigma-Aldrich, St. Louis, MO, USA) as the primary antibody for 2 h after imaging; the rest of the protocol followed the abovementioned.

A scanning laser densitometer (GS-700; Bio-Rad, Hercules, CA, USA) was used to evaluate each band’s density [optical density (OD)/mm^2^], which is shown as the mean ± standard error (SE). The density ratios of the pERK1/2 and total ERK1/2 bands to that of the β-actin band were compared using Molecular Analyst software (Bio-Rad, Hercules, CA, USA). One- or two-way analysis of variance with the Student–Newman–Keuls post hoc correction was used for statistical analysis, depending on the dataset analysed. A *P*-value of < 0.05 was considered indicative of statistical significance.

## RESULTS

### ERK1/2 expression in bull testes and epididymides

**Figure 1:**
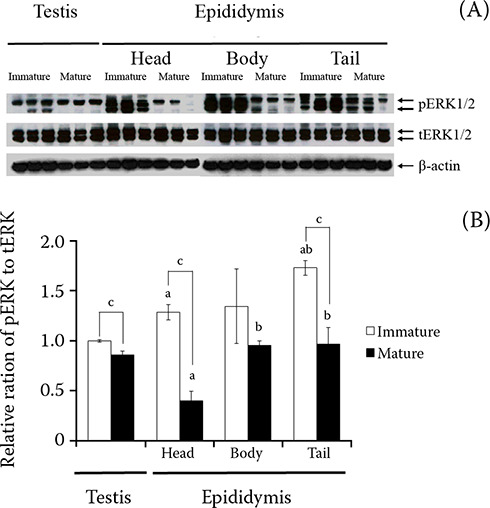
Western blot analysis of extracellular signal-regulated kinase (ERK, ~44/42 kDa) in the testis and epididymis of immature calves and mature bulls (A) Representative immunoblots for the detection of phosphorylated ERK (pERK; ~44/42 kDa), total ERK (tERK; ~44/42 kDa), and β-actin (~45 kDa). Arrowheads indicate immunoreactivity. (B) The activity of ERK was normalized to the signal for total ERK expression. Values for the testis of immature calves were arbitrarily defined as 1.0 *P < 0.05 vs. testis; **P < 0.05 vs. head of the epididymis; ***P < 0.05 vs. immature bull organ

To investigate the developmental expression level of pERK1/2 and total ERK1/2 (molecular weight ~42/44 kDa) in immature and mature bull reproductive organs, the testis and epididymis lysates from bulls were analysed using Western blotting (Figure 1). In immature bulls, the pERK1/2 activity in the testis was lower than that in the epididymis, whereas pERK1/2 activity increased from the head to the body and tail in the epididymis. However, the relative OD values for pERK in lysates demonstrated lower activity in the testis and all regions of the epididymis of mature bulls than in those of immature bulls.

In immature bulls, the relative OD value was higher in each region of the epididymis than in the testis. Similarly, in mature bulls, the relative OD value was generally higher in the epididymis than in the testis, except that the head of the epididymis had the lowest value. When comparing the expression by the epididymal region, ERK expression increased from the head to the body and to the tail in both immature and mature bulls.

### Immunohistochemical localisation of ERK1/2 in bull testes and epididymides

The results of pERK1/2 immunostaining in the testes and epididymides of mature and immature bulls are summarised in Table 1.

**Table 1:** Immunohistochemical staining for pERK1/2 in various cell types in the male reproductive system of healthy immature calves (5-month-old) and mature (24-month-old) bulls

Tissue	Cell type	pERK1/2	
		immature	mature
Testis	spermatogonia	++	+
	spermatocyte/spermatid	ND	+
	sustentacular cell	++	+
	sperm	ND	–
	myoid cell	–	–
	interstitial cell	++	+
			
Head of the epididymis	stereocilia	–	+
	principal cell	+	++
	basal cell	+	++
	sperm	ND	-
	connective tissue	++	++
			
Body of the epididymis	stereocilia	–	–
	principal cell	++	–
	basal cell	+	+
	sperm	ND	–
	connective tissue	+++	–
			
Tail of the epididymis	stereocilia	++	–
	principal cell	+++	–
	basal cell	++	–
	sperm	ND	–
	connective tissue	+++	+

Stained sections were scored for the density of positive cells per field

(−) = negative; (+) = weak; (++) = moderate; (+++) = intense; ND = not detected; pERK = phosphorylated extracellular signal-regulated kinase

**Figure 2:**
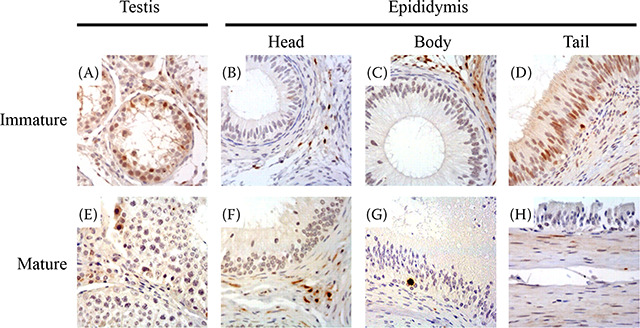
Immunohistochemical staining of extracellular signal-regulated kinase (ERK) phosphorylation in immature (A–D) and mature (E–H) bovine testis (A, E) and epididymis (B–D and F–H) (A, E) phosphorylated ERK-positive cells were observed in spermatogonia, sustentacular cells, interstitial cells, and interstitial cells in calves, whereas, in adults, immunoreactivities were noted in spermatogonia and interstitial cells. (B, F) In both calves and adults, positive reactions were observed in principal and basal cells in the head of the epididymis, although stronger reactions were observed in adults than in calves. (C, G) In the body of the epididymis, positive reactions were observed in principal and basal cells in immature calves, whereas reactive cells were found in only some basal cells in mature bulls. (D, H) In the tail of the epididymis, more than moderate reactions were observed in principal cells, basal cells, and stereocilia in immature calves, whereas these reactions were not detected in mature bulls

In both mature and immature testes, pERK1/2 was detected in spermatogonia, sustentacular (Sertoli) cells, and interstitial (Leydig) cells, but not in spermatocytes, spermatids, sperm, and peritubular myoid cells (Figure 2A,E).

In mature bulls, although strongly stained single cells in the testes were observed sometimes, the overall staining intensity was very faint and generally weaker than in immature bulls. pERK1/2 expression in the epididymides showed different patterns depending on the two age groups as well as the regions from which the tissues were obtained. First, in the head of the epididymis, some principal cells, basal cells, and surrounding connective tissues showed positive reactions in immature bulls; however, in mature bulls, in addition to the abovementioned cells, immunoreactivity was confirmed in the stereocilia and was generally stronger than those in immature bulls (Figure 2B,F).

The body of the epididymides showed stronger reactivity in immature bulls than in mature bulls, and immunoreactivity was confirmed in principal cells, basal cells, and connective tissues to varying degrees; however, only some basal cells were identified in mature bulls (Figure 2C,G).

In the tail of the epididymis, strong reactivity was observed in stereocilia, main cells, basal cells, and connective tissues in immature bulls, whereas reactivity was observed only in some connective tissues in mature bulls (Figure 2D,H).

## DISCUSSION

In this study, specimens of the male reproductive system collected from immature and mature bulls were examined to describe ERK1/2 expression using immunohistochemistry. Depending on the animal species, all mammalian testes mature in the same basic manner, which includes (1) prenatal formation of spermatic cords containing gonocytes; (2) transformation of gonocytes into spermatogonia; (3) profound sustentacular cell and spermatogonial proliferation; (4) maturation of sustentacular cells and formation of the blood–testis barrier; (5) development of spermatocytes (through meiosis); and (6) spermiogenesis (formation of round and elongated spermatids) (Picut et al. 2018). Sex steroids play very important roles in these processes although several studies have shown that the ERK cascade is involved in controlling steroidogenesis in steroid-producing cells (Cameron et al. 1996; Gyles et al. 2001; Seger et al. 2001). However, the findings from these studies seem to be in conflict as both stimulatory (Cameron et al. 1996; Das et al. 1996; Gyles et al. 2001) and inhibitory effects (Seger et al. 2001; Tajima et al. 2003) have been reported. Immunohistochemical staining revealed stronger ERK1/2 expression in the testes of immature bulls than in those of mature bulls in this study, and differences in expression between the two groups were confirmed depending on the region of the epididymis from which the specimens were obtained. In the epididymis, ERK1/2 expression increased from the head to the tail of the epididymis in immature bulls, whereas in mature bulls, immunoreactivity decreased from the head to the tail. As ERK is mechanically related to cell differentiation, this result is consistent with the tendency of ERK expression to increase at each site at the time of immature and mature development when sperm is produced and stored after maturation. Spermatogenesis in immature bulls starts as early as 16 weeks, and the first sign of its successful completion occurs at 32 weeks when elongated spermatids appear in the seminiferous tubules (Curtis and Amann 1981). Furthermore, according to Bagu et al. (2006), the average number of sustentacular cells per sperm continually increases from 13 to 33 weeks, as indifferent support cells differentiate into mature sustentacular cells in the testes. As 5-month-old animals were used in this experiment, it can be inferred that ERK expression is associated with the time when development is concentrated in the epididymis tail; moreover, the results for the testes suggest that ERK expression numerically increases with sustentacular cell maturation.

More studies should explore the correlation between hormones and ERK expression during the developmental processes and evaluate the concentration of testicular gonadotropin receptor concentration and serum LH, and the age of animals, such as neonates and very old animals, needs to be described more precisely.

In conclusion, we evaluated ERK expression in immature and mature bulls based on the sites in the testis and epididymis. Both age groups showed different ERK expression patterns and thereby confirmed ERK involvement in cell differentiation during maturation. To our knowledge, this is the first study to identify a role for ERK male reproductive system development in cattle.
